# A Novel Fingerprinting Technique for Data Storing and Sharing through Clouds

**DOI:** 10.3390/s21227647

**Published:** 2021-11-17

**Authors:** Mehvish Fatima, Muhammad Wasif Nisar, Junaid Rashid, Jungeun Kim, Muhammad Kamran, Amir Hussain

**Affiliations:** 1 Department of Computer Science, COMSATS University Islamabad, Wah Campus, Islamabad 47040, Pakistan; mehvishfatima01@gmail.com (M.F.); wasifnisar@gmail.com (M.W.N.); 2 Department of Computer Science and Engineering, Kongju National University, Cheonan 31080, Korea; 3 Department of Cyber Security, College of Computer Science and Engineering, University of Jeddah, Jeddah 21959, Saudi Arabia; mkkamran@uj.edu.sa; 4 Centre of AI and Data Science, Edinburgh Napier University, Edinburgh EH11 4DY, UK; a.hussain@napier.ac.uk

**Keywords:** cloud environment, secure signature, data usability, minimum distortion, robust fingerprinting

## Abstract

With the emerging growth of digital data in information systems, technology faces the challenge of knowledge prevention, ownership rights protection, security, and privacy measurement of valuable and sensitive data. On-demand availability of various data as services in a shared and automated environment has become a reality with the advent of cloud computing. The digital fingerprinting technique has been adopted as an effective solution to protect the copyright and privacy of digital properties from illegal distribution and identification of malicious traitors over the cloud. Furthermore, it is used to trace the unauthorized distribution and the user of multimedia content distributed through the cloud. In this paper, we propose a novel fingerprinting technique for the cloud environment to protect numeric attributes in relational databases for digital privacy management. The proposed solution with the novel fingerprinting scheme is robust and efficient. It can address challenges such as embedding secure data over the cloud, essential to secure relational databases. The proposed technique provides a decoding accuracy of 100%, 90%, and 40% for 10% to 30%, 40%, and 50% of deleted records.

## 1. Introduction

With the tremendous growth of digital content in recent decades, data sharing and distribution of digital content over the Internet have become a frequent practice in this digital age. On-demand availability of different services (i.e., hardware, software, and network-based services) brought revaluation in the IT industry. It becomes possible with the arrival of the concept of cloud computing. Cloud computing is an automated shared environment of configurable computing resources and has gained popularity in the last two decades [1]. It is an emerging paradigm of information processing storage and distribution to various nodes. Cloud enables the client to access the resource pooling when successfully executing the demand for services.

Cloud computing is revolutionizing the information technology industry by enabling access to their infrastructure and application services on a subscription basis. However, data copying and customizing data ability is like promoting intellectual property rights violation. At the present time, data security in the cloud environment is a demanding area of research. Different security measures enable the owner to secure his digital property, such as copyright protection, which is a form of intellectual property law that protects the original work of the author [2]. The technology faces the challenge of knowledge prevention, ownership right protection, security, privacy measurement of valuable and sensitive data because illegal copying, violation of ownership, and data burgled become a security issue. The data breach instance is caused by the inside thread, in which confidential and sensitive data has been viewed, stolen, and illegally distributed by a distinct individual by any organization. Data breaches may encompass professional secrets, health information (HI), personally identifiable information (PII), and other intellectual property. Unique customer identification marks are embedded in the data to detect illegitimate documents.

The identification of unique customer identification in terms of watermark data is known as fingerprinting $\left(F\right)$. This technique aims to protect the copyright and piracy of digital property from illegal distribution. It ensures effectiveness, particularly while passing out the process from attacker channels. Fingerprinting covers a wide variety of data formats used in various application areas [2,3,4,5,6,7,8,9]. This includes images audio [10,11] video [12], software [13,14], XML documents [9,15], geographic information system, relational database [8,16,17,18] etc. Fingerprinting ensures a security check to track the guilty traitor by embedding different fingerprints in each user relational database. Despite the growing popularity of cloud computing, security, privacy, and portability issues commonly exist in a relational database and other multimedia contents on the cloud environment. Security concerns are too demanding issues that create hurdles in the fast acceptance of the cloud for a relational database with a secure and shareable environment for on-demand service at the demand level of individual interests.

Digital fingerprinting is also called a digital signature. Digital signatures are used to demonstrate that a specific entity submits or approves specific data. Digital fingerprints are used to confirm that the data provided is correct. However, each copy of the document assigns a unique signature. It provides the highest security as a digital signature provides and is also useful in finding the guilty traitor.

This paper provides a concise analysis of the challenges facing the cloud community. These are affected online with the on-demand availability of the relational database. Moreover, it will show an effective approach to meet the existing challenges. In this paper, we proposed a novel fingerprinting technique for the cloud environment to protect numeric attributes in the relational database for digital privacy measurement. This fingerprinting scheme is capable of addressing security challenges for numerical data over the cloud. We presented a robust and efficient fingerprint scheme for a relational database capable of protecting and operating the database in real-time.

The rest of the paper is organized in the following sections. Section 2 discusses the existing fingerprinting techniques and their shortcomings. Section 3 discusses the research methodology, and Section 4 provides a systematic analysis of the proposed technique. Section 5 describes the attacker channel and the discussion, and Section 6 explains the comparison and analysis. Finally, Section 7 presents the experiments and the results with the overall conclusion in Section 8.

## 2. Related Work

With the rapid trend of cyber space usage, online accessibility of the relational database has become very common over the cloud environment. In [19], cloud base securities issues and vulnerabilities concerning cloud models were presented. The challenges to preventing sensitive data privacy issues are designed to ensure false insurance claims are handled in a manner authorized. Digital data may be in various formats like image, video, audio, natural language text, software, and relational database. These data types may expose to various types of attacks [8,18,20,21]. Agrawal [22] presented a classical algorithm of a watermarking primitive for relational data and proposed the bit-resetting algorithm. The proposed data scheme of the rightful owner is modified by embedding the selected bit in the least significant bit (LSB) ξ of the nominated attribute of the particular selected tuples. The selection of tuples is determined by computing some mathematical formulation with secret parameters. These secret parameters are only known to the true data owner. Secret parameters are encompassed using the candidate of attributes ν, least significant bits of nominated attribute *ξ*, a number of selected tuples *γ*, and fix length hexadecimal secret key $K_{s}$, respectively. The H-hash function is used to decide the least significant bit (LSB), mark a given numeric nominated attribute of the selected tuples and be based on message authentication codes (MAC).

An abstract model of a QoS aware provider framework based on queuing theory is given [23]. Regarding the service rate, the model provides an optimal solution achieved by the optimization method. In [24], the layered architecture of the IoT network is presented, as well as comparisons of the cloud-computing and edge-computing paradigms. By altering the framework of the IoT (internet of things) network, network attacks can be carried out without contacting network systems. Various distributed cloud computing frameworks offer apparent advantages for computing big data in real-time or offline, and cloud computing is fault-tolerant and dependent on high YARN cluster management. The goal of availability is to prevent the node from going down. As a result, cloud computing plays a critical role in gathering energy data [25]. Task scheduling methods [26] are used to manage resources and provide the best possible quality of service (QoS). Any network architecture, such as the virtual IT data center, must have security as the top priority. Somewhere on the edge, security is focused on protecting data center endpoints from external threats and ensuring a secure gateway to the Internet [27]. Additionally, the Secure Data Transfer Scheme (S-DTS) can handle the transmission of scattered data across many clouds [28]. Blockchain technology is used to enable trust in various clouds and is also used for secure data [29]. The proper and secure data transmission in the mobile cloud is used to overload traffic and improve security [30].

MAC [31] is designed as a specific type of hash function. It is used to verify the authenticity and integrity of the given data. It depended on a key based on the hexadecimal system with a selected tuple, a primary key, and a secret key, respectively. Data hiding in the least significant bit is an efficient system. However, an attacker may simply remove the fingerprint bits by rounding off the numeric attribute values very quickly. Note that an interesting fact about our proposed method is based on updating of bit values for each iteration performed. This approach successfully decodes the fingerprint system by updating the attribute values. In the paper [15], the authors proposed the usability for preserving the algorithms of query optimization and generating collusion secure fingerprint codes that support the usability constraints against several attacks. The collusion is secure for secret codes to choose from a precise codebook. However, it does not use the existing work against noise tolerance (several attacks), limited availability of anti-collusion codes, inadequate for large datasets, and external memory usage.

Cryptographic functions are used to generate secure fingerprint codes. Boneh and Shaw [20,32], Guth [33,34], and in [21], adopted the composition of codes using fingerprint The construction of collusion randomized secure codes in a restricted way [20,32]. First, they constructed a code matrix and performed some random permutations on the columns of this matrix to generate unique cubic length codes. The shortcoming of the listed technique created the collusion-secure codes. Meanwhile, our proposed approach generated fixed-length secure codes with MD5 hash function for multiple users in the cloud environment. We will generate a fingerprint string in real time for each client to generate a request. In [35,36], develop a reversible data technique is developed to hide in audio using a statically computed histogram and hidden key.

In [37], authors presented cloud base securities issues and vulnerabilities concerning cloud models. In [38], the researchers anticipated the investigation for security uncertainty in a relational database watermark tuple. Two security models of watermarking were demonstrated regarding the leakage of secret watermarking, which contents are single-key multiple databases (SKMD) and multiple-key single databases (MKSD). Clouds are an emerging paradigm of shared and automated environment, where data is stored on remote servers and available to Internet users [39]. Cloud computing aims to provide various computing resource applications as a service on demand, hardware infrastructure, software applications, and network services. Everything from data centers to demands on user applications can be consumed on a pay-per-use basis [40].

In [41], presented the fingerprinting framework is presented in a cloud environment to identify the guilty agent, to protect digital content from illegal distribution. When successfully logged in to their cloud account, an authentic and verified user generates the request query to access the protected data. An automated system invokes the fingerprint manager two modules, and the parameters manager performs the fingerprinting generation and embedding. When a request is submitted to access, this computerized system initiates the fingerprinting process and invokes the fingerprint manager to generate a string of fingerprint bits. Each client uses their private information and passes it to the parameter manager, where all necessary parameters are stored. The integrity of the users is verified by the manager module and checks the user authenticity. If the authentic user is found and requested to access the digital content, the fingerprint process will proceed; otherwise, the system will deny it. The secret signature from a fingerprinted relational database of the majority voting scheme is applied.

The proposed technique is really on the original database and parameter module due to relying on original data. This technique is not blind and always requires the original dataset for the authenticity of the user. Furthermore, our proposed technique is semi-blind. The authors in [15] proposed the algorithm for usability preservation of collusion-secure fingerprinting that supports several attack usability constraints against several attacks. Therefore, fewer watermark bits have been discovered. In [10], it is not space-efficient and increases the complexity of scalability enhancement. It does not work against noise tolerance. Limited anti-collusion codes are available and it is inefficacious for large datasets.

## 3. Proposed Methodology

We considered a relational database represented by $R_{\Delta }=\left(PK,\;A_{0},A_{1},\ldots ,A_{\left(n-1\right)}\right)$ in terms of the scheme data. Here, PK is named as a primary key and $\vartheta_{PK}$ attribute (in the absence of a primary key, an attribute is added to act as the unique attribute and auto-increment with the increment of new tuples) and $A_{i}\left(i=0,\ldots ,\;n-1\right)$ are the numeric attribute to nominate the required candidate for fingerprinting. Let there be n tuples in $R_{\Delta }$, a fraction 1/r of which will be used for the encoding of the fingerprint, F, where “r” is a user-defined parameter that is computed through the threshold formula given below. Since a robust watermarking scheme inevitably introduces small distortions to the data, it is assumed that each attribute value can tolerate modifications of at least least-significant bits (LSB) denoted with ξ. Table 1 describes all the parameters and notation.

**Figure 1:**
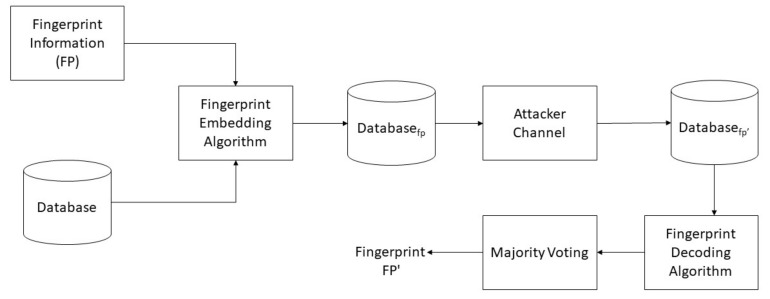
An overview of the proposed security model.

**Figure 2:**
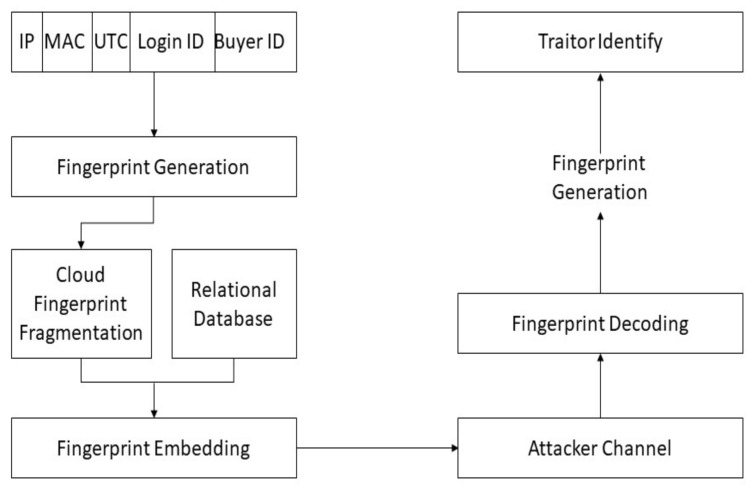
The sequential workflow of the approach.

Figure 1 demonstrates the proposed security model, and the block diagram summarizes all the core components of the fingerprinting technique. The *ξ* is a constant number and is independently used for an attribute value. Therefore, it depends on the number of binary digits of the attribute value. Figure 2 demonstrates the Sequential Workflow of the scheme in which fingerprint signatures are generated, embedded into the relational database. The proposed approach is used in a relational database for standalone systems, as well as in a relational database in the cloud. The traitor attack and decoding of fingerprints from the given database were used to identify the traitor. This block diagram summarizes all core components of the fingerprinting sequence of encoding decoding and traitor attack.

### 3.1. Cloud Computing Data Sharing Platform

Cloud computing is an automated shared environment. It provides a secure entrance for acquiring the restricted content with high authenticity. Private content is contained genuine information to access the desired digital information for secure purchasing to prohibit the distribution of the illegal copy of the digital information

This sensitive information may include important business documents. For example, a students’ result card, coupon numbers for a lottery, sales profit, income tax information, patient medical history, etc. This restricted content is used for those users who successfully cross the security and payment barrier, called a data-as-a-service. Moreover, data as a service provides on-demand and support to both data users and data providers at once. Microsoft Azure [42], Infochimps [43], Amazon [44], Oracle [45] are categorized as the most famous service provider to facilities the client at a low cost with 24/7 available and sustainability. Every service provider has its terms and conditions to provide access to needy users to obtain private content.

### 3.2. Security Model

**Figure 3:**
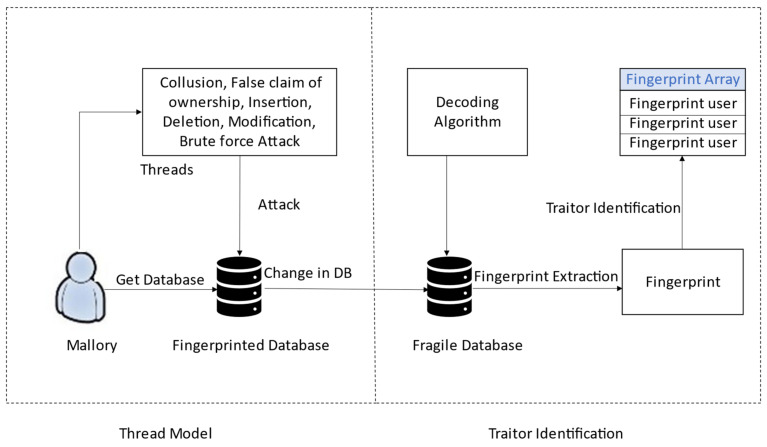
The security model.

Figure 3 shows the proposed security model. The proposed security model has thread and traitor identification.

#### 3.2.1. Attacks on Relational Database

A fingerprint/watermark can quickly destroy or remove from the marked databases. Some of the common attacks that Mallory can make on these threads in the cloud environment are shown in Figure 3. The Mallory has various attack vectors, such as insertion, deletion, modification, and Collusion Attacks. Mallory gets multiple copies and compares them with false claim ownership attacks and brute-force attacks. When these attacks are applied to the fingerprinted database, the attacks change the fingerprinted database to a fragile database. Then, Mallory tries to claim that this database is his database. After that, we take this fragile database, pass it from the attacker channel, decode the embedded fingerprint, and apply majority voting. Finally, we identify the guilty traitor.

#### 3.2.2. Collusion Attack

In this attack, Mallory may access various copies of the fingerprinted relational database, compare the differences among these copies, and remove all the signature values. The attacker applies the strategy of mix and match or majority counting to explode the fingerprint signals. This attack is specific to the fingerprint; all other attacks are related to both watermark and fingerprinting. Our proposed technique relies on this mix and match because some values do not change after embedding the fingerprint in selected rows. Mallory may skip these rows and be unable to perform the Collusion Attack. Our technique overcomes Collusion Attacks without losing the data quality.

#### 3.2.3. False Claim of Ownership

In this attack, the traitor seeks to provide fake evidence to claim the owners to pirate the false evidence. Mallory adds his new fingerprint/watermark signature and attempts to claim wrong ownership in this kind of attack. Inveracity attack occurs when the attacker randomly changes some fingerprinted bits value and discovers values are guessing successfully. Ours proposed technique is also resilient against this type of thread because we apply fingerprints to selected tuples. The selection of these tuples is made with a calculated formula. Mallory adds fake fingerprints to a database to claim ownership. However, when we pass this database from the thread model and decode fingerprints from the fragile database, we can identify the theft.

#### 3.2.4. Insertion Attack

In this insertion/additive attack, an attacker adds extra tuples into the datasets, assuming that the inserted signature is bothered, resulting in synchronization error. Moreover, Mallory considers the degradation of data quality and condition. However, it does not make any changes to the embedded fingerprint value in the relational database. We can decode the fingerprint with 100% decode accuracy and quickly identify the traitor.

#### 3.2.5. Deletion Attack

First, Mallory aims to delete a subset of data from the relational database. Then, he tries to remove the fingerprint from the embedded data to destroy the data quality and conditions. When we pass this fragile database from the threat layer, our decoding identifies a traitor if Mallory removes 95% subsets of data. However, our technique is still robust even against 95% record deletion attacks.

#### 3.2.6. Begin Updates

In this type of attack, some relational database processing performs unintentionally on fingerprinted/watermarked data. Therefore, Mallory adds some marked data deleted, inserted, alternated that may cause the fully or partially destroy the embedded watermark string. In our proposed scheme, a security key with secret parameters generates a combination of static and dynamic parameters. These parameters are IP/MAC addresses, UTC, login ID, and buyer ID. In addition, these parameters create different scenarios in real life. Therefore, Mallory does not guess the secret key and finds the exact tuple where the fingerprint has been embedded. However, our proposed technique is resilient against all kinds of modification and updates up to 95% of fingerprinted data.

#### 3.2.7. Brute Force Attack

In this attack, Mallory tries every possible combination to guess the security signature and think about the secret parameters such as the secret key. The attack is prevented by assuming that the size of the secret parameters is long enough. For generating secret keys, static and dynamic combinations of parameters are used and generated in real-time. Whenever the user logins to generate the fingerprint, some parameters change according to the situation. Eight real-time situations can occur while the user is generating the fingerprint. These parameters are the IP address of the user, MAC address of the systems, UTC the Coordinated Universal Time, Buyer id that generated at the time of buying request, and the login id that is unique for every user on the cloud. We generate unique fingerprint strings with a user-specific parameter when a customer generates a request to access the dataset. We get fix 35-bits unique identification signature as the string for each customer. Signature generation, through our proposed mechanism, has the ability that an attacker cannot generate and crack the existing signature. We have a 35-bit string length with the possibility of a 34,359,738,368 combination. It takes too much time for Mallory to guess exact bits or run the burst-force attack. Another strong motivation for robustness is that we have embedded the whole signature string into each nominated attribute.

### 3.3. Real-Time Scenarios for Fingerprint Bits Generation

Different scenarios are observed on real-time usage of a cloud environment for a signature generation with multiple parameters to specify the user authentication. These parameters include some private and public parameters. The private parameters have only legitimate users, and public parameters are accounted for any other user’s knowledge, like an email address for a login account on the cloud environment. Login account $J_{A}$ Password, IP/MAC addresses A, UTC τ, and buyer id β or (order ID ψ) assigns at the time, successfully placing the order. Different schemes could happen in real life when we consider the parameters mentioned above. Secure and private parameters are used that are only known to the data owner. These are a hexadecimal security key $k_{S}$, and cryptographic hash function H.

#### Framework for Fingerprint Bits Generation

**Figure 4:**
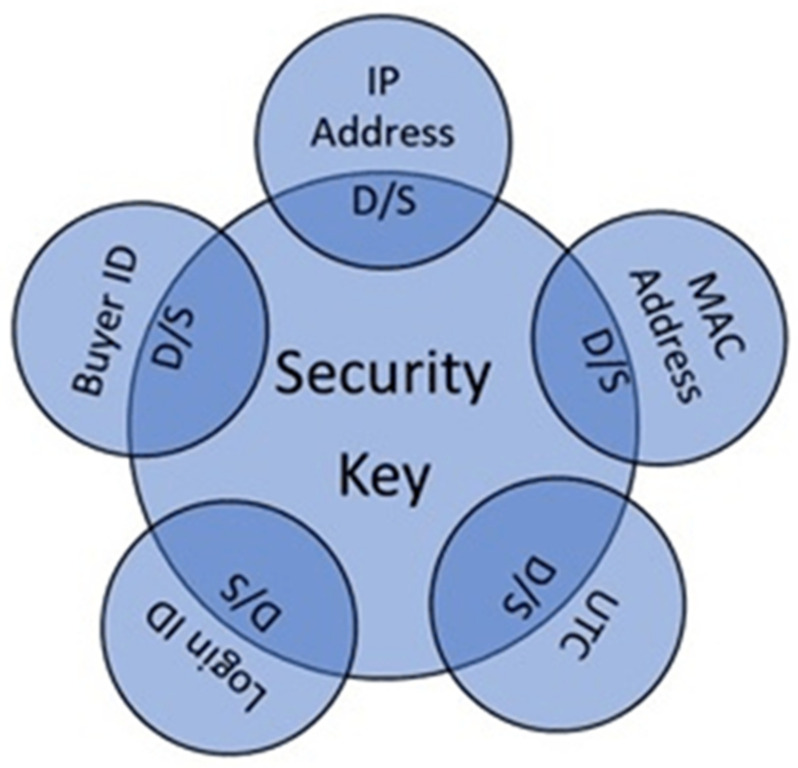
Framework for fingerprint bit generation.

Figure 4 shows the generation of a framework for the fingerprint bit generation.

Signature generation with multiple parameters to specify the user’s authentication is observed on a cloud environment real-time usage. These parameters include some private and public parameters. The private parameters are the constraints that the only legitimate user knows. Public parameters account for the knowledge of any other users or attackers, such as an email address for a login account on the cloud environment. Login account $J_{A}$ Password, IP/MAC addresses A, UTC τ, and buyer id β or (order ID ψ) assigns at the time, successfully placing the order. Different schemes could happen in real life when we consider the parameters mentioned above. The secure and private parameters are used that are only known to the data owner. These are a hexadecimal security key $k_{S}$, and cryptographic hash function H. Table 2 lists the parameter variations for generations of fingerprints.

As listed in Table 2, various parameters participate in the generation of the fingerprint. On the left side of the table, five parameters directly belong to the cloud user. These parameters have two necessary states. Either they are unique S or different O in their function.

Scenario 1: When a client has used a unique buyer id, same IP and MAC address but logs in at a different time and login ID are used, which can be formatted as follows:

$$≬\beta \left[S\left(A\left(Z\right)\wedge \;O\left(J_{A}\left(X\right)\right)\;\wedge \;O\left(\tau \left(Y\right)\right)\;\right)\;\right]$$

Scenario 2: A user can access a cloud environment through their unique cloud login account $J_{A}$ with unique MAC and static IP address A of his system, and then may order to get specific information (database) from this cloud. An individual buyer ID β is assigned against each request generated by using to acquire the database. This scenario can be formulated as follows:

$$≬\beta \left[S\left(A\left(Z\right)\;\wedge \;S\left(J_{A}\left(X\right)\right)\;\wedge \;O\left(\tau \left(Y\right)\right)\;\right)\;\right]$$

Scenario 3: There is a buyer ID with a different IP/MAC address but the same login time and login ID. It can be formulated as follows:

$$≬\beta \left[O\left(A\left(Z\right)\;\wedge \;S\left(J_{A}\left(X\right)\right)\;\wedge \;S\left(\tau \left(Y\right)\right)\;\right)\;\right]$$

Scenario 4: Some buyer id has a different IP, MAC address, and login ID and but a diverse period, which can be formulated as follows:

$$≬\beta \left[O\left(A\left(Z\right)\;\wedge \;O\left(J_{A}\left(X\right)\right)\;\wedge \;S\left(\tau \left(Y\right)\right)\;\right)\;\right]$$

Scenario 5: There is some buyer id, which opens with different IP, MAC address, and login ID but logs in simultaneously, which can be formulated as follows:

$$≬\beta [O(A\left(Z\right)\;\wedge \;O(JAƎb\left[D\left(add\left(Z\right)\;\wedge \;D\left(A\left(X\right)\right)\;\wedge \;S\left(T\left(Y\right)\right)\right)\right]$$

Scenario 6: There is some buyer id, which has different IP and different MAC address, login ID, and login time, which can be formulated as follows:

$$≬\beta \left[O\left(A\left(Z\right)\;\wedge \;O\left(J_{A}\left(X\right)\right)\;\wedge \;O\left(\tau \left(Y\right)\right)\;\right)\;\right]$$

Scenario 7: There is some buyer id that has the same IP and MAC address, login ID, and same login time, which can be formulated as follows:

$$≬\beta \left[S\left(A\left(Z\right)\;\wedge S\left(J_{A}\left(X\right)\right)\;\wedge S\left(\tau \left(Y\right)\right)\;\right)\;\right]$$

Scenario 8: Some buyer id has the same IP and MAC address and login time but different login ID, which can be formulated as follows:

$$≬\beta \left[S\left(A\left(Z\right)\;\wedge \;O\left(J_{A}\left(X\right)\right)\;\wedge \;S\left(\tau \left(Y\right)\right)\right)\right]$$

### 3.4. Logical Data Partition

Dataset D is divided into “m” nonoverlapping partition. This data partition does not change the data physical location; nevertheless, this data partition is based on logical grouping. Initially, the total numbers of subgroups are equal to the value of m and kept secret and only know the owner of the data to make the partition approach secure and resilient to the attacks. Finally, the subgroups are categorized into two major portioning bins, named even and odd partitioning bins. Even the $m^{\prime }$ partition contains only those candidates whom zero remainders. Finally, the odd partitioning bin contains those nominated candidates who attribute to partition number one remainder.

### 3.5. Threshold Computation

The threshold value is computed to select the candidate attributes from the selected usability constrained as an attribute. The threshold is calculated to shortlist the candidate attributes for marking the digital signature. The noise in the data is reduced and provides high security to the end-user. It made it more difficult for an attacker to guess the nominated attribute value. Moreover, an important advantage of using an appropriate threshold is that it minimizes the desired distortion in the data, and the authenticity of the data remains. We consider that there is a minor capability in that we can make a few minor data changes that do not affect the originality of the data. Only those tuples whose candidate attribute value is greater than the threshold value are selected to insert into the shortlisted subdata set as the original sub-data set. This partitioning is used as logical, not physical. All tuples are inserted into this database when the candidate attributes value satisfies the threshold value.

### 3.6. Fingerprint Embedding

Our fingerprinting technique embeds a robust multi-bit fingerprint string identifier for each client in the numeric attributes of the relational database nominated tuples. The robust fingerprinting scheme aims to protect the database from illegal distribution, ownership proofing, and resilience against a wide range of attacks with 100% decoding accuracy. At that time, there is only one fingerprint tuple left after the attack. Fingerprinting is used to insert unique bit code for each buyer, which creates the smallest distortion or noise in relational data. There is an assumption that database clients can endure a small amount of noise. The Fingerprinting database is delivered multiple copies to multiple buyers. We present a scheme that overcomes the resultant minimum distortion technique.

### 3.7. Fingerprint Decoding

Fingerprint decoding is used to decode the embedded fingerprint bits ${\tilde{F}}_{P}$ String from the suspicious data. Fingerprint detection is raised in a possible scenario when Alice (data owner) accused that some data sets are illegitimately copied, redistributed, or tampered from her relation dataset $R_{\Delta }$. Alice or a third party verifies the ownership of the suspicious database through a fingerprint detection algorithm. The fingerprint decoding efficiency of a fingerprinting algorithm usually depends on the small bandwidth; the larger the bandwidth, the better the decoding accuracy. In other words, more fingerprint techniques are required against multiple attacks and vice versa.

### 3.8. Majority Voting

The majority voting procedure is performed. It aims to improve security and decoding accuracy against a wide range of malicious attacks.

### 3.9. Robust Analysis for Fingerprinting Scheme

**Figure 5:**
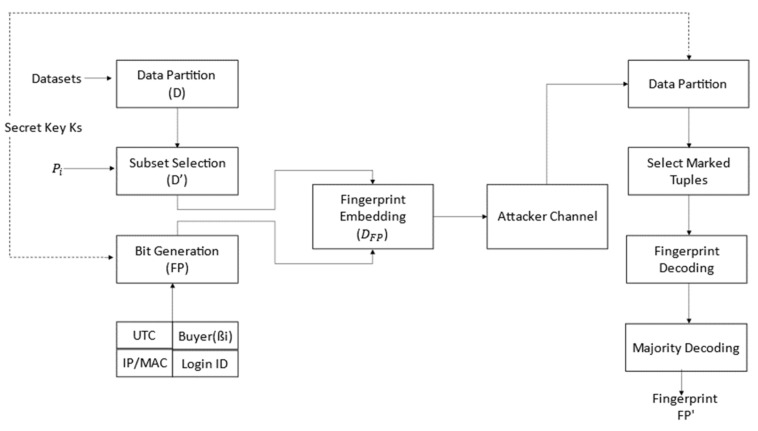
Aspect system architecture.

Robust fingerprinting schemes aim to preserve digital contents from illegal copy detection, ownership proof, or copyright. Figure 5 shows the architecture of the proposed system.

A robust fingerprint identifies and validates the buyer information embedded into a numeric attribute of the relational database. It carries the guilty traitor by matching the decoding string to an array of embedding strings. A bit string embedded as a fingerprint is a meaningful sequence generated through the proper sequence. A meaningful pattern is an image, owner information, or an audio signal. The meaningless sequence can be a random bit or computed through the client information at run time with some private attributes such as secret key $k_{S}$ and cryptographic hash functions MD5. These meaningless string sequences are robust and provide to strengthen the property of the desired fingerprint. The Meaningless bit string is unpredictable and robust against a wide range of malicious attacks such as insertion, deletion, and updating attacks. The main objective of malicious attacks is to remove, make undetectable, untraceable, tampering the relational database. When a fingerprint is embedded in digital content, it may make some noise and data distortion in the original content. It produces an overall change in relational databases compute using mean μ and standard deviation σ before embedding the fingerprint bits and after completing the signature embedding procedure to the database.

Our proposed fingerprinting technique focuses on minimizing the noise produced by embedding the fingerprinting and overall data distortion. It is assumed that the relational database can tolerate a small amount of noise, which causes data distortion. In [8], the robust watermarking technique is presented to change the nominated attribute with some percentage change of the actual original data value. However, this may produce more distortion in the original value.

On the other hand, our proposed robust fingerprinting technique can minimize the distortion generated by embedding the fingerprint bits into the relational database numeric attribute. Another drawback of the existing technique relies on the original content value of the selected attribute of original data. Our proposed approach introduces an array containing some values independent of the original data and only known to the data owner or data protection rights authority. This file observed size is extremely small compared to the original database. Another aspect related to the robustness of the fingerprinting technique is the total bandwidth size available to mark the fingerprinting. A larger bandwidth means that it has more possibilities and spaces to embed the fingerprint string. The least bandwidth means that there are limited choices for the insertion of fingerprint, and it is not much more robust against malicious attacks by the attacker. A high degree of robustness is achieved by increasing usability constraints and the size of the relational database bandwidth.

## 4. Systematic Analysis of the Proposed Approach

A robust fingerprint algorithm is used to embed fingerprint bits into the dataset. The fingerprint embedding algorithm takes a secret key $k_{S}$ and the fingerprint bits $F_{P}$ as an input and converts a dataset D into fingerprint dataset DFP. The modifications (distortions) made by watermarking are bounded by the usability constraints matrix χ. Our technique is defined only once for every possible type of application that will eventually use the dataset. The fingerprint encoding process can be summarized in the following steps:

### 4.1. Data Partition

To initialize the embedding process, we first divide our relational dataset into a number of subgroups. Where *m* is a private constraint number known to the data owner, these partitions are nonoverlapping, and division is performed logically. The physical data position [33] remains similar, and it does not affect the appearance of the original dataset. This dataset is divided into m (e.g., m =100) number of partitions, namely as $P_{i}$ = {$P_{0}$, $P_{1}$, …,${\;P}_{m-1}$,}. This logical division fulfills sets theory fundamental operations (e.g., union, intersection, and empty set). The intersection of different sets is observed empty because no common element lies in each partition. Each tuple belongs to only one partition as $S_{i}\cap S_{j}=\Phi$. It is also satisfied that the property of unions in which each partition is exhausted into the original dataset as $P_{0}\cup P_{1}\cup P_{2}\cup P_{3}\cup$ … $\cup P_{m-1}$ = D. This logical data partition holds the property that every sub-group contains at least one tuple in it. No data partition group can be empty as $P_{i}$ *=* Φ Several tuples in each partition are not necessary to be equal. Data partitioning was completed into two-level partitions. Data is partitioned formulated to categorize data into an “*m*” number of subcategories in level one. The final partition was then performed and every subgroup was categorized into two main categories for $m^{\prime }=0$ and $m^{\prime }=1$. In the relational database, the primary key of every tuple is concatenated with the secret key $k_{S}$ and a cryptographic hash function. The secret key is the private parameter and is only known to the data owner. Generates the hexadecimal string depending upon the hash function type. We use the MD5 (Message Digest 5) hash algorithm to provide extensive security and generate a 1a 28-bit hash value. After getting the hexadecimal value, the method value of m is considered. The value of m is also kept secret. It is categorized every row into m number of sub-groups. After completing the first level sub-grouping, divide every sub-group element into two levels of further partitioning. Algorithm 1 shows the data partition.
**Algorithm 1.** Data Partition**Input:** $R_{\Delta }$, $k_{S}$, P **Output:** Data partitions $P_{i=1\ldots m}$
1: for each $r\in R_{\Delta }$ do 2: Calculate tuple subset $r_{\Delta }\left(r\right)$← (H (r·ϑ|$k_{S}$)) mod m
3: Insert r into $P_{\Delta }\left(r\right)$
4: end for 5: return $P_{i}$


### 4.2. Selection of Subset Data

To minimize the distortion and noise in the original data, only selected tuples are nominated for fingerprint. The selection of attributes is based on usability constraints that the data owner defines. Usability constraints are used to control the usability of data loss, define by the merchants, and identify within the available bandwidth for fingerprint embedding. Bandwidth is the maximum capacity of a relational database where the data is located. The fingerprint decoding efficiency of a fingerprinting algorithm usually depends on this bandwidth; the larger the bandwidth, the better the decoding accuracy. In other words, the more fingerprint technique is more robust against multiple attacks and vice versa. The data recipient wants authentic and original data without any anomaly with minimum distortions. The merchant defines usability constraints to bind the alterations. To preserve the information, merchants define usability constraints in a relational database. Selected attribute constraints pick up based on the values that they contained. The resultant values perform increment or decrement operations on constrained attribute values. The mean and standard deviation of the final values did not change.

We compute the data selection threshold by the formula given in Equation (1) for attribute selection for selecting data for fingerprinting.

(1) $$\Gamma \;=\frac{\mu_{i}\times {\;\mu }_{i\;}\left(\rho \%\right)\;}{\sigma_{i}{}_{\;}\times \left(c\right)}$$

Data partitions ($P_{0}$, $P_{1}$, …,$P_{m-1}$,) are given as input for data selection and database ${\tilde{R}}_{\Delta }$ as the resultant output. Database ${\tilde{R}}_{\Delta }$ contains only tuples whose value satisfied the threshold limit. For the fingerprint encoding process, only those tuples will insert into the database ${\tilde{R}}_{\Delta },$ whose attribute Ai is above threshold Γ. For each partition tuple in $P_{0}$, $P_{1}$, …, $P_{m-1}$, mean (σ), some percentage value that is selected by choice of the user ρ% standard deviation (μ) and the percentage value of the standard deviation $\mu_{i}\rho$%. The percentage value lies 0 to 100 and is defined by the data owner. The c is the confidence factor with a value between 0 to 1. The percentage value of the standard deviation $\mu_{i}\rho$*%* and c are kept secret. Enhance security mechanisms make it very difficult for an attacker to guess the selected tuples in which the fingerprint is inserted. These values are randomly defined by the data owner. The purpose of using two secret parameters is unable to guess the values of both parameters percentage value of the standard deviation $\mu_{i}\left(\rho \%\right)$ and confidence factor c correctly. It becomes impossible in every aspect for the attacker to calculate the threshold. The standard deviation performs threshold computation $\mu_{i}$ multiply with the percentage of standard deviation $\mu_{i}\rho \%$ and divides with the resultant mean (σ) multiply with the value of confidence factor c. Threshold value obtained from equation two and all attribute values greater than threshold Γ are saving into the database ${\tilde{R}}_{\Delta }.$

(2) $${\tilde{R}}_{\Delta }\leftarrow {\;rA}_{i}>\Gamma \;$$

Fingerprint embedding is performed on these nominated attributes of selected tuples in the database ${\tilde{R}}_{\Delta }$ and $rA_{i}$ are attributes for selected tuples. There is a presentation of the nominated attributes of selected tuples. They contain the attribute values from $A_{i}$, 0 to n − 1 tuples and attributes columns and v referred to the value of these attributes $R_{0}$ as described in Equation (3).

$$As{\;A}_{0}=v_{00},{\;v}_{10},\ldots ,v_{\left(n-1\right)0}\;$$

$$A_{1}=v_{01},{\;v}_{11},\ldots ,v_{\left(n-1\right)1}\ldots$$

$$A_{\left(n-1\right)}=v_{0\left(n-1\right)},{\;v}_{1\left(n-1\right)},\ldots ,v_{\left(n-1\right)\left(n-1\right)}\;$$

The selected tuples $R_{i}$ as follows:

$$\;\;\;\;\;\;\;\;\;R_{0}=v_{00},{\;v}_{01},\ldots ,v_{0\left(n-1\right)}{\;R}_{1}=v_{10},{\;v}_{11},\;\ldots ,v_{\left(n-1\right)1}\;\ldots {\;R}_{\left(n-1\right)}$$

(3) $$R_{0}=v_{00},{\;v}_{01},\ldots ,v_{0\left(n-1\right)}{\;R}_{1}={\;v}_{\left(n-1\right)0},{\;v}_{\left(n-1\right)1}\ldots ,v_{\left(n-1\right)\left(n-1\right)}$$

Algorithm 2 depicts the different steps of this phase.
**Algorithm 2.** Data selection**Input:**${\tilde{R}}_{\Delta }$ Data partitions $P_{0}$, $P_{1}$, $\ldots ,\;P_{m-1}$, 1: for i = 0 to m − 1 do 2:  for each $rA_{i}\;\in \;P_{i}$_,_ do 3: Compute μ and σ on $A_{𝒾}$
4:    Compute $\left(\rho \%\right)$ value of μ
5:    Calculate Γ using Equation (1) 6:  end for 7: end for 8: return ${\tilde{R}}_{\Delta }$←$rA_{i}>\Gamma$
**Output:** ${\tilde{R}}_{\Delta }$


### 4.3. Fingerprint Bits Generation

In this step, the F fingerprint bits are generated for each client when access requests are submitted in the cloud environment. Binary fingerprint strings are generated from client indication information based on IP address, MAC address A, date-time stamp UTC (Coordinated Universal Time) τ, login ID $J_{A}$, and buyer ID β. These constraints are obtained in real time. Thus, Fingerprint string generation is calculated with the lowing function given below:

$$F_{P}\leftarrow \left(k_{S},\beta ,A,\tau ,J_{A}\right)=F_{P}$$

where “|” specifies the concatenation operation perform among buyer ID $\beta_{i}$, IP MAC address, UTC date-time stamp τ, login ID $J_{A}$ with secret key $k_{S}$ and a cryptographic MD5 hash function to generate the fingerprint bits $\left\{f_{𝓅0}f_{𝓅1}f_{𝓅2}\ldots f_{𝓅\left(L-1\right)}\right\}$ as shown in Equation (4).

(4) $$F_{P}=f_{𝓅0}\;f_{𝓅1}f_{𝓅2\;}\;\ldots \ldots f_{𝓅\left(L-1\right)}$$

These bits are given as input to the fingerprint encoding function. The length of the fingerprint is L, where L is the fixed-length 128-bit has to value $F_{P}$ generated by the MD5 hash function.

$$F=\overset{L-1}{\underset{𝒾=0}{\sum }}f_{𝓅\left(𝒾\right)}\;$$

Fingerprint generating function ℷ transform the hexadecimal (alphanumeric) string into a binary bit string $\left\{f_{𝓅0}f_{𝓅1}f_{𝓅2}\ldots f_{𝓅\left(L-1\right)}\right\}$.

$$ℷ\Lambda :\;f_{𝓅\left(𝒾\right)}\to \;F_{P}$$

However, ℷΛ this entails keeping all buyer-fingerprint codeword string $f_{𝓅\left(𝒾\right)}$ in a database $F_{P}$ for decoding persistence. It leads to security overhead by keeping the fingerprint codes database secret to ruin a collision attack. Some security measures are essential to be enforced thus as to protect the database access control, password protection, and encryption. To overcome the security issues, the data owner generates a unique buyer’s fingerprint $F_{P}$ from the merchant’s secret key $k_{S}$ and the buyer’s series number $\beta_{i}$ (which can be public/private) using a cryptographic hash function H. A function H maps the data of arbitrary size string “S” to data of fixed size string “𝒽” and the values returned by the hash function are called the hashes. Algorithm 3 shows the fingerprint generation.

H:S→𝒽

**Algorithm 3.** Fingerprint generation**Input:**$k_{S}$, β, IP, A, τ, and $J_{A}$ **Output:**F 1: for i = i to 𝓃  do 2: $f_{𝓅\left(𝒾\right)}=H\left(\tau ||\left[H(k_{S}\left|\left(A\right)\right||\left(H\left(k_{S}|\beta \right)\right)\right]\;\right)$ 3: Insert $f_{𝓅\left(𝒾\right)}$ into F 4: end for 5: return F

### 4.4. Fingerprint Embedding

The Fingerprinting algorithm uses the multi-bit algorithm. Multibit means that for each client, the length of the fingerprint is 0 to L-1 and embeds this bit string in the selected tuples of the nominated attributes of a database ${\tilde{R}}_{\Delta }$. For the sake of simplicity, we are assuming that partition set $P_{i}$ in database ${\tilde{R}}_{\Delta }$ contains signal attribute $v_{i}∊P_{i}$. Our fingerprinting technique embeds a robust multibit fingerprint signature identifier for each client in the numeric attributes of the nominated tuples of the relational database. For signature embedding XOR $\left(⊕\right)$ Operation is performed between the fingerprint signature string and m. The fingerprint signature string is generated through algorithm two, and m is the logical partition number of level two. It computes the resultant bit. The result will be one of the sign bits, and the m bits are the same; otherwise, it will be zero. This step executes for L-1 several times, which is the actual length of the fingerprint signature. All collective bits are combined and stored in a resultant array $\hat{F}$. The length of this array is also equivalent to the fingerprint signature string. The resultant array preserves bits string for each buyer in it, and this array is kept by data owner or copyright monitoring authorities.

$$f:\;(D,\;FP)\to F_{\Delta }$$

The general encoding rule is defined in Equation (5).

(5) $$f_{i}=u_{i}\;+\;v_{i}{fb}_{i}$$

Here, $f_{i}$ represents the fingerprinting coefficient equivalent to the original coefficient $u_{i}$, $v_{i}$ denotes the fingerprinting strength, and $fb_{i}$ denotes the portion of the fingerprint signature.

Total data distortion that makes throughout the fingerprinting procedure, as

$$\Delta \leftarrow ({\tilde{R}}_{\Delta }-R_{\Delta })$$

Suppose the resultant array is the same as the least significant bit LSB of the nominated attribute for multi-bit fingerprint embedding. In that case, LSB ξ keeps remaining the same otherwise flip. Eliminate the least significant bit (LSB) after encoding and recheck the remaining bits in the fingerprint signature string. When there is zero left and halt embedding. Repeat this step for L-1 times and update the attribute value. Multibit fingerprint signature length is 0 to L-1 and computed collusion-free fixed-length signature string in a real-time scenario using Algorithm 2. Bit m will remain the same for a full fingerprint signature, as it is generated for the nominated attribute based on the primary key of the selected attribute. An identical fingerprint signature is applied to all nominated attributes of the selected tuple. Repeat the same applied procedure for all data partitions $P_{i}$. It improved the protection level if an attacker successfully deleted or updated the fingerprinted attribute value and left only one fingerprinted attribute. Our proposed approach provides 100% decoding accuracy. Sometimes, it is critical in real life that data statistics do not contain anomalies. It may also require sensitive data that statistics such as mean and standard deviation resultant should equal or approximately, before and after the fingerprint signature embedding. Algorithm 4 explains fingerprint embedding.

$$\mu \left(R_{\Delta }\right)\;≅\;\mu \left(\hat{F}\right)$$

$$\sigma \left(R_{\Delta }\right)\;≅\;\sigma \left(\hat{F}\right)$$

**Algorithm 4.** Fingerprint embedding**Input:**$k_{S}$, $R_{\Delta }$, ${\tilde{R}}_{\Delta }$, β, F, Length of fingerprint L **Output:** $F_{\Delta }$, $\hat{F}$ 1: for each row r in $P_{𝒾}$ do 2: Calculate Γ using Equation (1) 3: ${\tilde{R}}_{\Delta }\;\leftarrow \;rA_{i}>\Gamma \;$
4: Generate $f_{𝓅\left(𝒾\right)}\to \;F$ using Equation (2) 5: for each r in ${\tilde{R}}_{\Delta }$
6: compute $m^{\prime }$ bit 7: $\;m^{\prime }$ = 0 if $𝓅\left(𝒾\right)\;\%2=0$
8: otherwise $m^{\prime }$=1 9: $K_{𝒾}=m^{\prime }\;⊕\;f_{𝓅\left(𝒾\right)}$
10: compute $\hat{F}$←$K_{𝒾}$
11: end for 12: SET $K_{𝒾}$ to *LSB*
ξ of ${\mathrm{rA}}_{i}$, where $K_{𝒾}=L$
13. end for 14. Return $F_{\Delta }$, $\hat{F}$

### 4.5. Fingerprint Decoding

Fingerprint decoding decodes the embedded fingerprint bits string from suspicious data sets that are illegitimately copied. The data partitions are generated using the same steps to perform fingerprint encoding to generate a data partition algorithm as in the fingerprint encoding phase.

#### 4.5.1. Decoding Strategies

Fingerprint decoding can be public or private, depending on security requirements. A blind decoding algorithm does not require an embedded fingerprint or any part of the original data. The marked attribute(s) and tuple(s) are identified, and this submodule decodes fingerprint bits. The fingerprint decoding phase detects fingerprint string from a suspicious database to identify the traitor user. For detecting, the same preprocessing step and formulations are performed to start the decoding process. These prepossessing steps are the same as a repeat in embedding phase, logical data partitioning, threshold computation, selection of nominated attribute, calculate the value of m two-way partition, selection of string from the resultant array, and length of the resultant array $F_{P}.$ XOR function is performed between the resultant array and m and computes fingerprint bits. Fingerprint bits are generated the same as the length 0 to L-1 and this step is repeated L times. Suppose that the resultant bit and m bit are the same and the generated fingerprint bit is equal to the least significant bit of the attribute value. In that case, there is no change in the least significant value of the nominated attribute. Otherwise, flip the least significant bit of the nominated attribute. Store fingerprint bit ${𝒻}_{𝓅\left(𝒾\right)}$ in an array ${\tilde{F}}_{Pi}$. When ${𝒻}_{𝓅\left(𝒾\right)}$ value is 0, make increment in the figure of zero else one.

#### 4.5.2. Majority Voting

To decode the final embedded fingerprint information appropriately and efficiently majority voting mechanism is performed. It aims to make better security and decoding accuracy against a wide range of malicious attacks. In the end, majority voting is performed and that the number of zero in the array ${\tilde{F}}_{Pi}$ are greater than one was verified, then set ${\tilde{F}}_{Pi}$ a value equal to 0; otherwise, one. Match the detected fingerprint string with the stored fingerprint and return the traitor information, the guilty user, and distribute illegal copies of the database. Fingerprint decoding is used to decode the embedded fingerprint bits string from the suspicious data. Fingerprint detection is raised in a possible scenario when the data owner is accused that some datasets are illegitimately copied, redistributed, or tampered from her relation data R. The fingerprint decoding efficiency of a fingerprinting algorithm usually depends on this bandwidth. The larger the bandwidth, the better the decoding accuracy. In other words, more fingerprint techniques make them more robust against multiple attacks. Algorithm 5 describes fingerprint decoding.
**Algorithm 5.** Fingerprint decoding**Input:** Fingerprinted Database $D_{FP}$, Secret Key $k_{S}$, Resultant array $F_{P}$ **Output:** Decoded Fingerprint $\tilde{F}$
1: Ones = 0, Zeros = 0
2: for each partition ${𝓅}_{𝒾}$, do 3: ${\tilde{R}}_{\Delta }\leftarrow$ Calculate Γ
4: compute $m^{\prime }$ bit for each $rA_{i}$
5: for 𝒾 = 0 to L−1
6:  for each row r in $\tilde{D}$, do 7:   ${𝒻}_{𝓅\left(𝒾\right)}=$ $m^{\prime }\;⊕\;K_{𝒾}$
8:     if (($K_{𝒾}==0\;\&\&\;m^{\prime }==0$) ∥ ($K_{𝒾}==1\;\&\&\;m^{\prime }==1$)) 9:       if ($\xi =={𝒻}_{𝓅\left(𝒾\right)}$) 10:     No change 11:      else 12:       Flip ξ bit 13:   ${\tilde{F}}_{Pi}\leftarrow {𝒻}_{𝓅\left(𝒾\right)}$
14: If ${\tilde{F}}_{Pi}=0$ 15:    Zeros=Zeros+1
16: Else Ones=Ones+1
17: end for 18: If(Zeros>Ones)
19:  $\tilde{F_{𝒾}}\;{\tilde{F}}_{Pi}=0$
20: else 21: ${\tilde{F}}_{Pi}\tilde{F_{𝒾}}=1$
22: $\tilde{F}\leftarrow \tilde{F_{i}}\;{\tilde{F}}_{Pi}$
23: end for 24: Return $\tilde{F}$


## 5. Attacker Channel and Discussion

A fingerprinting technique must have the capability that it is unable to locate by the intruder and significantly degrade the quality of data if the intruder tries to corrupt the fingerprint. Our scheme’s fingerprints are detectable and robust against different types of attacks generated on the fingerprinted data, including Collusion Attacks. We demonstrate the robustness of our scheme under a wide range of descriptive attacks. Among those attacks, some are common to fingerprinting and watermarking, while Collusion Attacks are unique to fingerprinting. Our proposed solution is vulnerable to a collision attack in which buyers collaboratively compare their data copies, disrupt the underlying fingerprints, identify differences of embedded marks, and modify their fingerprints. Our proposed scheme is resilient against various attacks, especially Collusion Attacks and minimum distortions in the original dataset. As shown in [22], the embedded marks may be ruined by making significant modifications to marked data. Therefore, the robustness of our scheme is considered relative to the data modifications made by attacks and updates. We analyze our fingerprinting scheme using the following robustness measures. We say that a binary string is a valid fingerprint if it is of the form F(Ks|βi) (0 ≤ *n* < βi). A detected fingerprint is incorrect if it is not the one that was used in the insertion of the fingerprint.

Now, we judge the effectiveness of our proposed scheme against a wide variety of attacks. Many attacks are common in watermarking and fingerprinting, such as intersection, deletion, and updating, and some are only related to the fingerprinting as a Collusion Attack.

The attacker inserts some new tuples at any place in a relational database to affect the existing fingerprint mark in the relation database $R_{\Delta }$, also called blind insertion. Inserting new tuples is used to disturb the sequence of embedded fingerprint bits that produce a synchronization error. We overwhelmed this synchronization error by using the primary key. Whenever new tuples are inserted, the auto-numbers series is followed throughout the relational record. Our fingerprint embedding scheme is implemented through partitioning of primary keys and logical data based on secret parameters such as secret key ks and the number of partitions m. A primary key cannot contain a repeating value and cannot be null. Therefore, it ensures that the order of tuples does not disturb the embedded fingerprint and the insertion of new tuples based on the primary key. The addition of new tuples increases the probability of false-positive rates. Insertion of non-fingerprinted new tuples can result in an increasing amount of false-positive rate. The insertion attack is also attacked to break the fingerprint structure of the embedded fingerprint. However, our proposed approach is resilient to the insertion attack. We embedded a full string in every partition. If only one tuple is left, our proposal can decode efficiently. Every tuple must have the primary key. So, the insertion of new records does not affect our fingerprinted data. The decoding accuracy remains 100% after inserting some new tuples.

Collusion Attacks belong to fingerprinting and consider an active attack. Malicious purchasers may collude to compare their fingerprinted versions. When multiple fingerprinted copies are distributed, Fingerprinting schemes are exposed to Collusion Attacks. In which attacker colitis, several fingerprinted copies of the document with a different signature. The associate peoples of a coalition may create a good data copy that does not implicate any coalition member. During fingerprint detection, either the copy may yield an innocent buyer fingerprint, or it may not yield a valid fingerprint. The collision attack is specific to fingerprinting, and there is no Collusion Attack in the watermarking setting. Resistance to Collusion Attacks can be critical in a fingerprinting application

By locating the positions where the documents differ, one can discover where the fingerprint bits are inserted. Modifying documents on these positions may obtain a new version without a readable fingerprinted document, escaping detection. For example, an attacker can open copies in the cloud environment to obtain any number of differently fingerprinted copies. Our proposed scheme uses multiple constraints for the generation of fingerprints and inserts through the hash function. The hash function is used to identify the attribute for each row where it inserts a fingerprint mark. We use bit generation Algorithm 2 to generate the attribute sequence to be marked for each buyer. As a result, the insertion pattern is randomized for each buyer. Hence, the fingerprint copies for different buyers of the same databases are so different that they cannot collude to find the insertion places of fingerprint marks.

Sometimes this attack is called a subset attack. This attack adversary aims to destroy the embedded fingerprint by selecting and deleting some tuples, either randomly or selected based on some statistical operations of attribute values, from the fingerprinted dataset DFP. The decoding algorithm task is to recover the fingerprint from the remaining tuples in each partition. Suppose adversary drops *N* tuples from Alice’s fingerprinted dataset [22]. We are assuming that the attacker does not know the portioning position of the tuples where the fingerprint was embedded, and also has not accessed the embedding secret key $K_{s}$.

In such an attack, the modification value of the data destroys the embedded fingerprinted marks. Attackers modify the value by flipping bits, rounding off the value, and alternating subset and superset. Attackers can modify the value by flipping bits and rounding off the value, subset, and superset alterations. Modification of values is performed the same as the usual data modification of data. However, more modifications mean more distortion in data. In crucial and other sensitive data, there is not much flexibility to accept the changes. Otherwise, the data will be useless. Modifying the selected fingerprint data increases the chances that attackers achieve their goal and successfully destroy the fingerprinted information. However, in our proposed technique, we use some secret parameters that make it difficult for an attacker to guess the exact nominated attribute. Bit attacker is based on a random selection of tuple attributes to change the bit value. There is also the possibility that the attacker changes the randomly selected bits to zero, also called zero-out the attack. Round-off the numeric data to remove the embedded signature is a kind of attack. The attacker seems to remove the signature bit with whole data and round-off the value. To make this attack effective, the attacker must estimate the correct figure of how many bits are involved. Data become useless and destroy data usability when the attacker overestimates the value. Underestimated has no significant effect on data and the embedded signatures.

## 6. Comparison and Analysis

This section shows the resilience against different types of attacks discussed in this research. In a deletion attack, the fingerprint attackers destroy the inserted fingerprint from the provided dataset by deleting some records. A fingerprint architecture protects relational data against duplication and transmission [41]. It guarantees that a user can only access data that contains their unique identifier. When a user accesses a database, the fingerprint process is activated, according to the technique. An administrator module performs fingerprinting activities using multiple fingerprinting functions, but these functions are vulnerable and change data values, making it difficult to identify a complicit agent because the administration component also verifies the authentication using these specifications. In [19] implies that if a change is made to the database for the insertion of messages, the normal interpretation of the data will not be interrupted. However, to account for distortion-based methods of watermark interpretability, consider distortion constraints.

**Figure 6:**
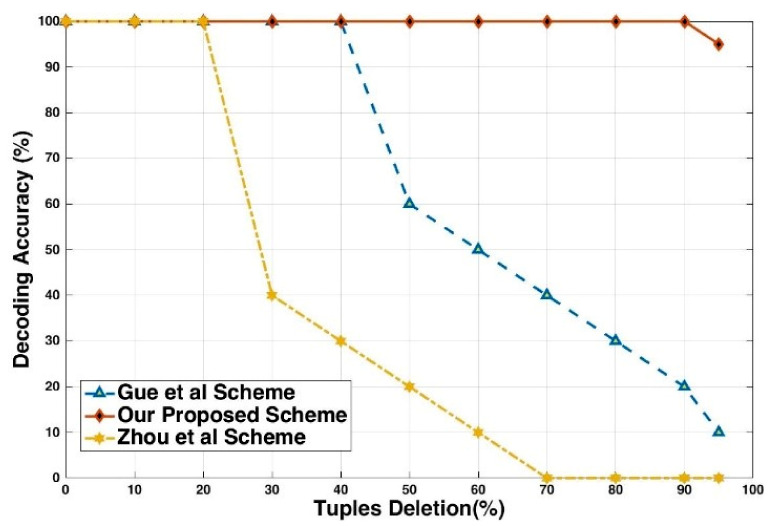
Decoding accuracy versus deletion of tuples.

The proposed technique results are higher against Zhou [41] and Gue [19] on resilience to this attack. The 95% of the tuples are deleted, and the fingerprint is ultimately detected. The attacker erases the embedded fingerprints and deletes more than 95% of the fingerprinted relation, which means he will lose almost the whole association. We conclude that this attack is not sufficient for the attacker. Suppose that the attacker examines each tuple independently and selects it with a probability pd for inclusion from the pirated relation. For the attack to be successful, it must delete all embedded bits for at least one fingerprint bit. We know that each fingerprint bit is embedded multiple times, so the probability that all the embedded bits for fingerprint(*fp*) are deleted. Figure 6 shows the result of decoding accuracy from the fingerprinted relation obtained by randomly selecting and deleting different ratios of tuples for the simulation of this attack.

**Figure 7:**
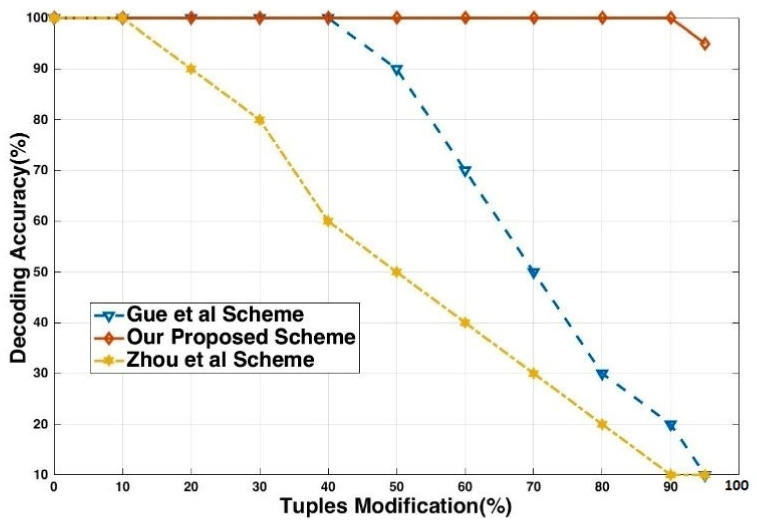
Decoding accuracy vs. tuples modification.

Figure 7 shows the decoding accuracy of the fingerprinted relation obtained by randomly selecting different ratios of the fingerprinted relation and toggling the least significant bits (LSB) for all their attributes and modifying LSB with other closest values with some increment or decrement. The 95% of the tuples are randomly altered, the fingerprint can be detected completely. It shows that for the attacker to erase the embedded fingerprints, they should alter more than 95% of the fingerprinted relation, causing a perceptible change to the pirated relation. In the majority voting, fingerprint detection does not detect fingerprint bit (*fp*). Therefore, the probability of the fingerprint bit is not recovered.

**Figure 8:**
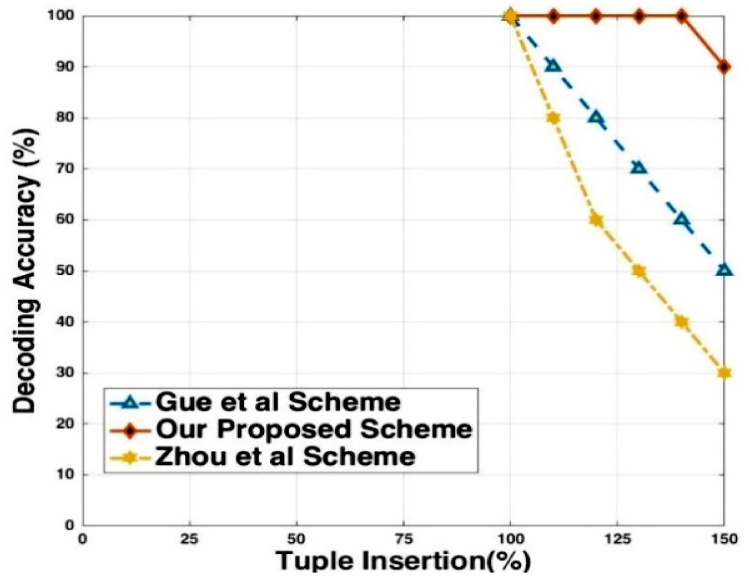
Decoding accuracy vs. tuples insertion.

A fingerprinting scheme should be robust against malicious attacks. However, the effect of these updates on the fingerprint detection process is limited to the minimum. It should be made hard for the attacker to modify the embedded fingerprint so that the innocent owner will not need to claim his ownership rights of relation, nor will they be considered a pirate. Figure 8 shows the decoding accuracy of the fingerprinted relation obtained by randomly selecting different ratios of the fingerprinted relation, mixing them with tuples from other sources, and inserting them into a relational database. We can see that the detection rate is approximately 90% when the attacker modifies 50% of the embedded fingerprints.

## 7. Experiments and Results

This section performed some experiments and analyzed the results to validate the robustness of our proposed technique. All experiments are performed on the SQL server 10.0 on Windows 10 PC with Intel (R) Core (TM) i3-2310M CPU 2.10 GH, 10 GB of RAM, and 500 GB hard drive. The C# is used as a programming language, and Dropbox as a cloud environment. The proposed approach is easily utilized for any relational database. Our approach does not depend on the size and nature of the database because the database is logically divided and its size does not matter. We obtained a dataset of liver disorders from the UCI directory [46]. We selected 10,000 numbers of tuples and seven attributes. We shortlisted the fingerprinting length of 35 bits binary string from a variable-length string. Another total number of partitions m = 100. The percentage change of value 𝓅% is 37% (1 to 100), and c is the confidence factor is 0.004 (0 to 1). Moreover, the threshold value is calculated through the threshold formula, and only the optimal value selects that value is greater than the calculated threshold. Our proposed technique is resilient against various attacks and demonstrates through experiments and malicious databases the ability to identify guilty traitors.

### 7.1. Analysis of Attacks

#### 7.1.1. Deletion Attack

**Figure 9:**
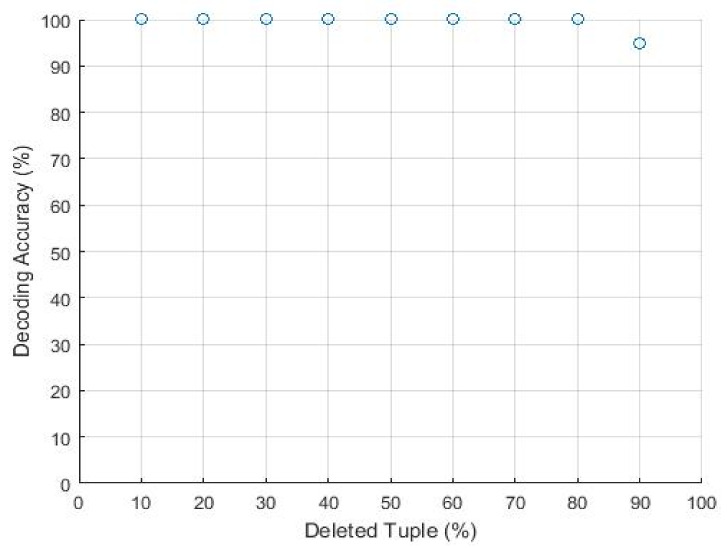
Modification deletion attack.

In this attack, the adversary drops some tuples intending to disturb the structure and sequence of embedded fingerprints in the relational dataset. The attacker accomplishes the tuple deletion mission to remove the fingerprint bits. It depends either on some statistical calculation or randomly picked attributes. For example, suppose the attacker drops a ℵ number of tuples from the Alice dataset and change ℵ number of values every time to analyze the effect on decoding accuracy. Figure 9 shows the decoding accuracy with the threshold base [1] fingerprinting technique. Our technique provides 100% decoding accuracy when the attacker drops more than 95% of the tuples. Moreover, when only one marked tuple is left in any partition of the relational database. Figure 9 shows the modification deletion attack and circle represent the accuracy percentage. 

Furthermore, our approach is not dependent on any mark value of tuple. We had presented the selection and picking of tuples on logical partitioning, and all those values are selected whose value is greater than the computed value of the threshold. This value selection and shortlisting mechanism for tuple make it more effective and resilient against synchronization error. Furthermore, we utilized the primary key to overwhelming this kind of errors. Our finding analysis for robustness is observed when the attacker dispels an extremely high tuple drop attack that affects the value of mean μ and standard deviation σ. Our technique protects against such a scenario, as our proposed technique decoding accuracy is independent of usability constraints.

#### 7.1.2. Collusion Attack

In this attack, the attacker obtains multiple copies of the fingerprinted document and tracks the difference between these documents. The attacker attempts to prove his ownership by destroying the embedded fingerprint and inserting his signature to claim ownership. However, our fingerprint signature generation mechanism is secure and robust against Collusion Attacks. This attack is specific to fingerprinting. We generate unique fingerprint strings with a user-specific parameter when a customer generates a request to access the dataset. We obtained a 35-bit unique identification signature for each customer’s string. Signature generation, through our proposed mechanism, has the ability that an attacker cannot generate and creak the existing signature. We obtained 35-bits string length 34,359,738,368 combination. The attacker does not guess the exact bits or run the burst-force attack. Another strong motivation again is robustness, that we have embedded the whole signature string into each nominated attribute.

#### 7.1.3. Modification Attack

**Figure 10:**
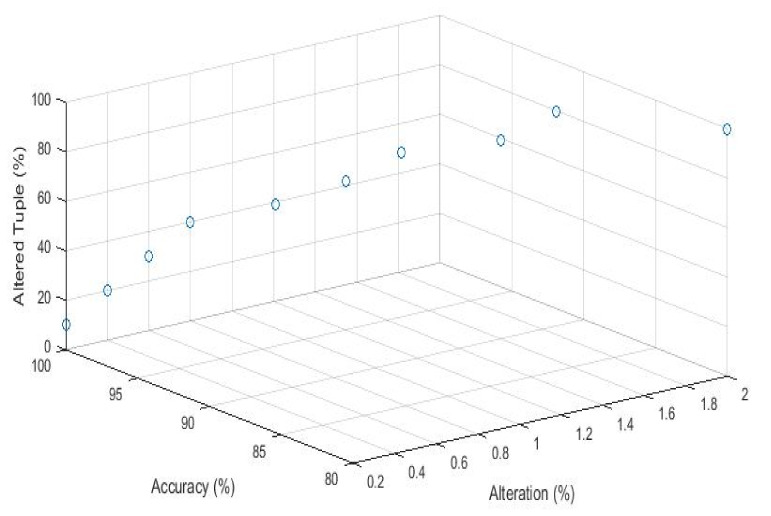
Modification attack.

The traitor modified the attribute value to destroy the embedded fingerprint signature. Our technique can decode these signatures with 100% decoding accuracy because our approach does not depend on the attribute value. Meanwhile, the techniques [19] depend on attribute values and decoding accuracy offended when the attacker achieves the goal of finding and updating all the fingerprinted attributes in the fingerprinted document. Figure 10 shows the modification attack and small circle showing the percentage of accuracy. 

#### 7.1.4. Insertion Attack

**Figure 11:**
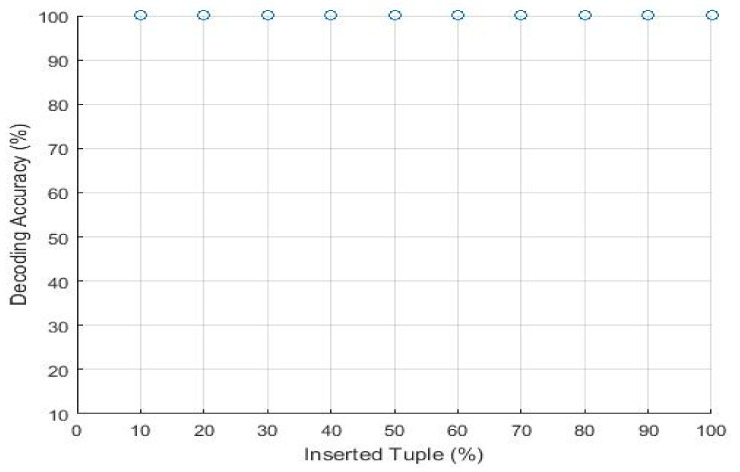
Modification insertion attack.

The attacker inserts new tuples to make the data distorted and noisy. There are several ways in which an attacker inserts the new tuple into the fingerprinted document. For example, there is a possibility that he inserts a new tuple at the end of the document. He might also insert a few tuples in random locations and duplicate the existing tuple in the relational database. Figure 11 shows the modification insertion attacks. These small circles represent the percentage accuracy. Our proposed technique is resilient for each type of insertion. We embedded the fingerprint signature based on the primary key and logical partitioning of the relational database. When an attacker inserts a new tuple with any insertion mechanism he used, a unique primary key will be assigned to each tuple. It is the properties of a primary key that cannot be duplicated or contain a null value. Our proposed approach decodes the signature with 100% accuracy when the attacker doubles the volume of the dataset.

## 8. Conclusions

This paper proposed an effective, reliable, and automated solution to protect precious numeric relational data in the cloud for illegal duplications, traitor identification, and redistributions. We presented a robust fingerprinting technique for relational databases with minimum distortion in the original database and bandwidth-independent decoding accuracy. We provided a unique signature generation mechanism for each buyer in real-time. The fingerprint embedding process is used to embed an arbitrary bit string as a fingerprint pattern using the secret key and the cryptographic hash function. The fingerprint decoding algorithm extracts the embedded bit string of the fingerprint. Our Proposed fingerprinting algorithm is highly resilient against various attacks; Insertion, Deletion, Alteration, Bit-flipping attack, and Collusion Attack, and presents a blind reversible scheme. The proposed scheme provides the database owner ownership authorization and attains full recovery of the original database relation once the fingerprint is detected and authenticated. We can tackle the combination of multibit fingerprints with collusion-resistant code generated through the buyer’s ID. Our fingerprinting scheme’s nature is asymmetric and provides a unique on-demand copy of data to requested buyers. Merchant uses the same identification marks for the detection process; to prove to a third party that the data is pirated and that the pirated data is sold to a specific buyer.

Further research should also investigate categorical, alphanumeric attributes, and non-numeric encoding domains, a logical extension of this research.

## Figures and Tables

**Table 1:** Parameters and notation.

Symbol	Description
$R_{\Delta }$	Relational Database
$F_{\Delta }$	Fingerprinted Data
ϑ	The primary Key value for tuple r
$rA_{i}$	The numeric attributes Ai of tuple r, where i=0 to n−1
r	Tuples
F	The fingerprint, represent as a string of length *l*
$\tilde{F}$	Decoded Fingerprint
$\hat{r}$	The fraction of tuples that are selected for fingerprinting
H	Hash function
$\tilde{H}$	Cryptographic hash function Message Digest (MD5)
$k_{S}$	Secret key
λ	Number of Buyers
L	Length of fingerprint
$\Delta_{i}$	Subset
β	Buyer ID
*η*	Number of partitions
ξ	Least significant bit (LSB)
Γ	Threshold
$J_{A}$	Login account
∁	Similar constraints
ψ	Different constraints
τ	Login time (UTC)
A	IP and MAC address
μ	Mean (Avg)
σ	Standard deviation
m	Number of partitions
$m^{\prime }$	*M* = 0 or *m* = 1
K	*K* bit
𝓅	Partition
$\tilde{D}$	Slash Dataset

**Table 2:** Variations in parameters for fingerprint generation.

Parameter	1	2	3	4	5	6	7	8
Buyer ID	D	S	D	D	S	S	D	D
IP Address	S	S	D	D	D	D	S	S
MAC Address	S	S	D	D	D	D	S	S
UTC	D	D	S	D	S	D	S	S
Login ID	S	S	S	S	D	D	S	D

D = Different, S = Same.

## Data Availability

Data sharing is not applicable to this article as the authors have used publicly available datasets whose details are included in the “experimental results and discussions” Section 5 and Section 7 of this article. Please contact the authors for further requests.

## References

[1] Mell P., Grance T. (2011). The NIST Definition of Cloud Computing.

[2] Muhammad N., Bibi N., Qasim I., Jahangir A., Mahmood Z. (2018). Digital watermarking using Hall property image decomposition method. Pattern Anal. Appl..

[3] Wang Y., Doherty J.F., van Dyck R.E. A watermarking algorithm for fingerprinting intelligence images. Proceedings of the Conference on Information Sciences and Systems.

[4] Kim J., Oh K., Oh B.S., Lin Z., Toh K.A. (2019). A line feature extraction method for finger-knuckle-print verification. Cognitive Computation.

[5] Nagra J., Thomborson C., Collberg C. (2002). A functional taxonomy for software watermarking. Australian Computer Science Communications.

[6] Storozhenko A. (2013). An Approach for Software Protection in Cloud Computing Environment Based on Watermarking Technique. Proceedings of the Computer Science & Engineering 2013 (CSE-2013).

[7] Sonnleitner R.G. (2016). Widmer, Robust quad-based audio fingerprinting. IEEE/ACM Trans. Audio Speech Lang. Process..

[8] Kamran M., Suhail S., Farooq M. (2013). A robust, distortion minimizing technique for watermarking relational databases using once-for-all usability constraints. IEEE Trans. Knowl. Data Eng..

[9] Wen Q., Wang Y., Li P. (2018). Two Zero-Watermark methods for XML documents. J. Real-Time Image Process..

[10] Haitsma J., Kalker T. (2002). A highly robust audio fingerprinting system. Ismir.

[11] Ramalingam A., Krishnan S. (2006). Gaussian mixture modeling of short-time Fourier transform features for audio fingerprinting. IEEE Trans. Inf. Forensics Secur..

[12] Neogi R. (2008). Fingerprinting Digital Video for Rights Management in Networks. U.S. Patent.

[13] Ma H., Ma H., Li R., Yu X., Jia C., Gao D. (2016). Integrated Software Fingerprinting via Neural-Network-Based Control Flow Obfuscation. IEEE Trans. Inf. Forensics Secur..

[14] Luo Y., Wang B., Wang X., Zhang B. (2016). SoftMF: A Software Defined Moving Fingerprinting Framework for Proactive Security Policies Enforcement. DEStech Trans. Eng. Technol. Res..

[15] Constantin C., Gross-Amblard D., Guerrouani M. Watermill: An optimized fingerprinting system for highly constrained data. Proceedings of the 7th Workshop on Multimedia and Security.

[16] Liu S., Wang S., Deng R.H., Shao W.A. (2004). A block oriented fingerprinting scheme in relational database. International Conference on Information Security and Cryptology.

[17] Li Y., Swarup V., Jajodia S. (2005). Fingerprinting relational databases: Schemes and specialties. IEEE Trans. Dependable Secur. Comput..

[18] Al-Haj A., Odeh A., Masadeh S. (2010). Copyright protection of relational database systems. Netw. Digit. Technol..

[19] Guo F., Wang J., Li D. (2006). Fingerprinting relational databases. Proceedings of the 2006 ACM Symposium on Applied Computing.

[20] Faundez-Zanuy M., Fierrez J., Ferrer M.A., Diaz M., Tolosana R., Plamondon R. (2020). Handwriting biometrics: Applications and future trends in e-security and e-health. Cognitive Computation.

[21] Nuida K. (2012). Short collusion-secure fingerprint codes against three pirates. Int. J. Inf. Secur..

[22] Agrawal R., Kiernan J. Watermarking relational databases. Proceedings of the 28th International Conference on Very Large Data Bases.

[23] Luo Y., Yang K., Tang Q., Zhang J., Li P., Qiu S. (2016). An optimal data service providing framework in cloud radio access network. EURASIP J. Wirel. Commun. Netw..

[24] Kaur K., Sharma S., Kahlon K.S. (2020). A Middleware for Polyglot Persistence and Data Portability of Big Data PaaS Cloud Applications. CMC-Comput. Mater. Contin..

[25] Chen J., Li K., Tang Z., Bilal K., Yu S., Weng C., Li K. (2016). A parallel random forest algorithm for big data in a spark cloud computing environment. IEEE Trans. Parallel Distrib. Syst..

[26] Pandey V., Saini P. (2019). Application layer scheduling in cloud: Fundamentals, review and research directions. Comput. Syst. Sci. Eng..

[27] Balakrishnan S., Surendran D. (2020). Secure Information Access Strategy for a Virtual Data Centre. Comput. Syst. Sci. Eng..

[28] Kim D.-Y., Min S.D., Kim S. (2019). A DPN (Delegated Proof of Node) mechanism for secure data transmission in IoT services. CMC Comput. Mater. Contin..

[29] Wang J., Chen W., Wang L., Sherratt R.S., Alfarraj O., Tolba A. (2020). Data secure storage mechanism of sensor networks based on blockchain. CMC-Comput. Mater. Contin..

[30] Sivakumar N.R., Ghorashi S., Jamjoom M., Alduailij M. (2020). Heuristic Bent Key Exchange Secured Energy Efficient Data Transaction for Traffic Offloading in Mobile Cloud. CMC-Comput. Mater. Contin.

[31] Paar C., Pelzl J. (2010). Message Authentication Codes (MACs). Understanding Cryptography: A Textbook for Students and Practitioners.

[32] Boneh D., Shaw J. (1995). Collusion-secure fingerprinting for digital data. Annual International Cryptology Conference.

[33] Guth H.-J., Pfitzmann B. (1999). Error-and Collusion-Secure Fingerprinting for Digital Data.

[34] Tardos G. (2008). Optimal probabilistic fingerprint codes. J. ACM.

[35] Liang X., Xiang S. (2020). Robust reversible audio watermarking based on high-order difference statistics. Signal Process..

[36] Wang W., Wang W. (2020). New High Capacity Reversible Data Hiding Using the Second-Order Difference Shifting. IEEE Access.

[37] Iqbal S., Laiha M.K., Babak D., Hussain M., Suleman K., Khan M.K., Choo K.-K.R. (2016). On cloud security attacks: A taxonomy and intrusion detection and prevention as a service. J. Netw. Comput. Appl..

[38] Lafaye J. An analysis of database watermarking security. Information Assurance and Security, Proceedings of the Third International Symposium on Information Assurance and Security.

[39] Armbrust M., Fox A., Griffith R., Joseph A.D., Katz R.H., Konwinski A., Lee G., Patterson D.A., Rabkin A., Stoica I. (2009). Above the Clouds: A Berkeley View of Cloud Computing.

[40] Sun Y., Zhang J., Xiong Y., Zhu G. (2014). Data security and privacy in cloud computing. Int. J. Distrib. Sens. Netw..

[41] Zhou M., Zhou M., Wang J., Wang C., Li D. A novel fingerprinting architecture for relational data. Proceedings of the 2007 Inaugural IEEE-IES Digital EcoSystems and Technologies Conference.

[42] https://azure.microsoft.com/.

[43] https://www.infochimps.com/.

[44] https://www.amazon.com/.

[45] https://www.oracle.com/cloud/daas.html.

[46] McDermott J., Forsyth R.S. (2016). Diagnosing a disorder in a classification benchmark. Pattern Recognit. Lett..

